# Association between driving pressure and recruitment-to-inflation ratio in personalized PEEP management at the bedside

**DOI:** 10.1038/s41598-026-36300-z

**Published:** 2026-01-19

**Authors:** Mert Yetgın, Hale Yetgın, Hülya Sungurtekın

**Affiliations:** 1 Department of Anesthesiology and Reanimation, Faculty of Medicine, Pamukkale University, Denizli, Turkey; 2 Department of Histology and Embryology, Faculty of Medicine, Pamukkale University, Denizli, Turkey

**Keywords:** Mechanical ventilator, Functional residual capacity, PEEP, Recruitment, Intensive care units, Health care, Medical research, Physiology

## Abstract

Positive end-expiratory pressure (PEEP) titration is crucial for preserving lung mechanics and physiology in mechanically ventilated patients. End-expiratory lung volume (EELV) and compliance can be used for this purpose. Based on these parameters, the Recruitment/Inflation ratio (R/I) has been used to estimate lung recruitability. We aimed to investigate the relationship between driving pressure and the Recruitment/Inflation ratio at two consecutive PEEP levels for appropriate PEEP selection in patients receiving mechanical ventilation support. End-expiratory lung volume (EELV) was measured during a decremental PEEP trial (PEEP levels of 20,15,10 and 5 cmH_2_O). The PEEP level associated with the lowest driving pressure among the four tested PEEP levels was selected. Patients were then ventilated at this selected PEEP level for 24 h. Subsequently, using the collected data, the R/I ratio was calculated between consecutive PEEP levels. If the R/I ratio was equal or above a pre-defined cut-off value, the higher of the two consecutive PEEP levels was considered potentially recruiting. If the R/I ratio was below the cut-off value, the lower PEEP level was considered appropriate. Finally, the relationship between the PEEP level associated with the lower driving pressure (between two consecutive PEEP steps) and the PEEP level deemed appropriate based on the R/I ratio cut-off was examined. The highest value of the arterial partial pressure of oxygen to fraction of inspired oxygen ratio (PaO_2_/FiO_2_) was measured at the 20 cmH_2_O PEEP level. EELV was found to be higher in patients classified as high recruiters (Step C and Step A) (*P* = 0.033). The PaO_2_/FiO_2_ ratio was also higher in the high-recruiter Step C (*P* = 0.008). In our study, the relationship between driving pressure and the R/I ratio for appropriate PEEP selection has been determined. The agreement between PEEP selection guided by driving pressure and PEEP selection guided by the R/I ratio did not reach statistical significance in our study. Sufficient and robust evidence to support the standalone use of the R/I ratio in clinical practice has not yet been established. Further studies are needed for this purpose.

**Supplementary Information:**

The online version contains supplementary material available at 10.1038/s41598-026-36300-z.

## Background

Functional Residual Capacity (FRC) is variable in mechanically ventilated patients in intensive care units (ICUs)^1^. FRC measurement, Positive End-Expiratory Pressure (PEEP), and alveolar recruitment maneuvers can be used to guide these therapies^2^.

A simplified modified nitrogen multiple-breath washout (NMBW) technique has been developed for End-expiratory lung volume (EELV) measurement^3^. EELV measurement can be used to determine optimal settings within the mechanical ventilation strategy. However, it is not sufficient on its own. It should be evaluated in conjunction with compliance^4^. The ΔEELV/ΔPEEP ratio, calculated across different PEEP levels, serves as a guide for selecting the optimal PEEP when interpreted alongside respiratory system compliance; here, ΔEELV denotes the mathematical difference in EELV between two PEEP levels. This is likely because the PEEP level associated with the highest compliance tends to coincide with the PEEP level at which the ΔEELV/ΔPEEP ratio is maximal^5^. The increase in EELV level with PEEP may be due to alveolar recruitment or alveolar distension. Therefore, EELV should be optimized not in isolation, but in conjunction with compliance^4^. Another parameter, driving pressure, is the difference between the airway inspiratory plateau pressure and PEEP^6^. That driving pressure approximates the tidal volume relative to the functional lung size, under the assumption of relatively constant specific compliance. However, because compliance is not constant—particularly at low and high lung volumes—driving pressure may not always directly reflect functional lung size^7^. At high PEEP levels, EELV alone does not reliably indicate lung distension. However, the Recruitment/Inflation ratio (R/I ratio), which accounts for changes in EELV relative to PEEP and compliance, can potentially be used for this purpose. The estimated recruited volume (referred to as ‘V estimated’) in the lungs is determined by subtracting the product of compliance at the lower PEEP level and the difference between the two PEEP values from the difference in EELV measured between these two PEEP levels^8^.

In a study by Chen et al.^8^, the R/I ratio was associated with both oxygenation and alveolar dead space. This association supports the validity of the ratio. If the R/I ratio is higher than the cut-off value, lung recruitment potential is considered high. Conversely, if it is lower than the cut-off value, the higher PEEP level carries a potential for overdistension. Many studies have used the median of the calculated R/I ratios (within their cohorts) as the cut-off value^9–12^.

There are a limited number of studies in the literature and clinical practice that utilize the R/I ratio as a standalone parameter. To our knowledge, the relationship between the R/I ratio and driving pressure for guiding appropriate PEEP selection has not been reported in the literature. In our study, we aimed to investigate the relationship between driving pressure and the Recruitment/Inflation ratio at two consecutive PEEP levels for appropriate PEEP selection in ICU patients.

## Materials and methods

Data for this study were obtained between July 2023 and July 2024 from 30 patients (aged 18–86 years) who were intubated for mechanical ventilation at the Intensive Care Unit of Pamukkale University Hospital, Turkey. All patients were non-ARDS cases, most commonly postoperative, neurological, or sepsis-related indications for ventilation (Fig. S1). All patients and/or their legal representatives provided consent to participate, agreed to the publication of the findings, and signed a written informed consent form prior to enrollment. This study was approved by the Non-Interventional Clinical Research Ethics Committee of Pamukkale University (Chairperson: Prof. Dr. Hülya Çetin, protocol number: 192.168.182.17, approval date: 13 June 2023). The study was conducted in accordance with the ethical principles for medical research involving human subjects outlined in the Declaration of Helsinki (1975, revised 2013).

Exclusion criteria were as follows: Patients with severe cardiovascular instability (mean arterial pressure < 60 mmHg, heart rate < 45 or > 150 beats/min), patients with pneumothorax, patients who had undergone pneumonectomy, patients with severe airway obstruction due to Chronic Obstructive Pulmonary Disease, patients managed on Continuous Positive Airway Pressure mode or with a T-piece, pregnancy.

Patients included in the study were connected to a CARESCAPE R860 (GE Healthcare) mechanical ventilator. To eliminate spontaneous respiratory effort, an intravenous loading dose of 0.6–1 mg/kg rocuronium bromide was administered. For sedation, an intravenous infusion of remifentanil was initiated with 1.5 mcg/kg followed by a continuous infusion rate of 0.5–15 mcg/kg/h. Patients were ventilated in volume-controlled ventilation (VCV) mode. Positive end-expiratory pressure was initially set to zero cmH_2_O. Tidal volume was set at 6 mL/kg based on predicted body weight (PBW). Respiratory rate was adjusted to achieve normocarbia (e.g., target PaCO_2_ 35–45 mmHg) based on arterial blood gas analysis, and the fraction of inspired oxygen (FiO_2_) was adjusted to maintain arterial partial pressure of oxygen (PaO_2_) between 80 and 100 mmHg. During the subsequent 15-min equilibration, FiO_2_ remained constant due to device software limitations. An end-inspiratory pause of 20% and an inspiratory-to-expiratory ratio of 1:2 were set. An ECOV-X (GE Healthcare) module for metabolic gas measurements was connected to the ventilator and allowed to warm up. A Y-piece spirometry kit and a heat and moisture exchanger (HME) were inserted into the ventilator circuit. Once a stable state was achieved in the patients, a PEEP titration procedure using the PEEP INview feature (Carescape R860, GE Healthcare, USA) was initiated. A baseline arterial blood gas (ABG) sample was drawn before starting the procedure. AutoPEEP was evaluated using the expiratory hold maneuver for 3 s at the end of expiration at zero end-expiratory pressure. None of the patients demonstrated measurable autoPEEP during the procedure. For standardization, a recruitment maneuver was performed by applying PEEP at 20 cmH_2_O for 30 s. A decremental PEEP trial was then conducted using four PEEP levels (20,15,10 and 5 cmH_2_O). Patients were maintained at each PEEP level for 15 min. At the end of each 15-min period at a given PEEP level, arterial blood gas analysis. static respiratory system compliance (Crs) using an end-inspiratory hold maneuver, tidal volume, peak inspiratory pressure (PIP), plateau pressure (Pplat), and driving pressure (DP = Pplat - PEEP) were performed and recorded. For safety reasons, EELV measurements were performed once at each PEEP level to avoid excessive derecruitment and hemodynamic disturbance.

The difference in EELV between two consecutive PEEP levels (e.g., 20–15 or 15–10 cmH_2_O) was calculated and denoted as ΔEELV. The estimated recruited volume (‘V estimated’) was obtained by mathematically subtracting the product of the difference between the two PEEP levels (ΔPEEP) and the compliance measured at the lower PEEP level ΔEELV. This estimated recruited volume (V_Est) was then normalized by dividing it by the ΔPEEP (C_Est). In fact, C_Est represents the compliance of the recruited lung tissue. To obtain the R/I ratio, the ' C_Est’ value was divided by the compliance value measured at the lower PEEP level^8^.

In order of calculation:

$${\mathrm{V}}\_{\text{Est }}\left( {{\mathrm{ml}}} \right) = \Delta {\mathrm{EELV}}{-}\left( {\Delta {\text{PEEP }} \times {\text{ Complicance}}_{{{\text{PEEP low}}}} } \right)$$

$${\mathrm{C}}\_{\mathrm{Est}} = {\mathrm{V}}\_{\text{Est }}\left( {{\mathrm{ml}}} \right)/\Delta {\mathrm{PEEP}}$$

$${\mathrm{R}}/{\mathrm{I}} = {\mathrm{C}}\_{\mathrm{Est}}/{\mathrm{Complicance}}_{{{\text{PEEP low}}}}$$

To clarify the relationship between our measures: the ratio ΔEELV/ΔPEEP represents the total compliance associated with the tested PEEP step (i.e., the sum of baseline compliance at the lower PEEP level and the compliance attributable to newly recruited/deformable lung units). We derived the recruited-lung compliance (C_Est) as:

$${\mathrm{C}}\_{\mathrm{Est}} = \left( {\Delta {\mathrm{EELV}}/\Delta {\mathrm{PEEP}}} \right) - {\mathrm{C}}\_{\mathrm{low}}$$

where C_low is the static compliance measured at the lower PEEP level. Thus C_Est quantifies the compliance of the recruited component, whereas ΔEELV/ΔPEEP corresponds to the total compliance measured across the PEEP step.

The PEEP level corresponding to the lowest driving pressure among the tested levels (20,15,10 and 5 cmH_2_O) was selected. If the minimum driving pressure was observed at more than one PEEP level, the lowest of these PEEP levels was chosen (to minimize the risk of barotrauma). Patients were subsequently ventilated at this selected PEEP level for 24 h. A Supplementary Fig. S1 has been added to illustrate the decremental PEEP protocol and R/I ratio calculation for improved reproducibility.

Based on the data recorded during the measurement phase, the cut-off value was defined as the median R/I rate calculated across all transitions for the 30 patients. When the R/I rate calculated between two consecutive PEEP levels was equal to or exceeded this cut-off value, the higher of the two PEEP levels was interpreted as having recruitment potential (high recruiter). Conversely, if the R/I rate was lower than the cut-off value, this was interpreted as indicating minimal recruitment potential (low recruiter) between these two levels, suggesting the lower PEEP level was adequate for that transition.

The primary outcome was the agreement between PEEP selection guided by driving pressure and R/I ratio; the secondary outcome was the relationship of the R/I ratio with compliance, oxygenation, and EELV (Table 1).

**Table 1:** Baseline characteristics and pre-maneuver lung mechanics of included patients (*n* = 30).

Category	Variable	Value
Demographic characteristics	Male sex, n (%)	17 (57%)
	Female sex, n (%)	13 (43%)
	Age, years	69.5 (22) [54–76]
	Height, cm	172.5 (12) [163–175]
	Body mass index, kg/m^2^	25.45 (2.88) [24.5-27.38]
Severity scores	APACHE II score	22 (6) [18–24]
	SOFA score	5 (2) [4–6]
Clinical outcomes	Mortality within first 72 h, n (%)	1 (3%)
Pre-maneuver lung mechanics	Static compliance, mL/cmH_2_O	56 (39) [36–75]
	PaO_2_/FiO_2_ ratio	300 (113.5) [249.5–363]
	Driving pressure, cmH_2_O	10.5 (9) [7–18]
	End-expiratory lung volume, mL	1074.5 (1915) [585–2500]
Primary indication for intubation	Postoperative, n (%)	12 (40%)
	Neurological, n (%)	10 (33%)
	Sepsis-related, n (%)	8 (27%)

Values are presented as median (interquartile range) [IQR: 25th–75th percentiles] or number (%). APACHE II = Acute Physiology and Chronic Health Evaluation II; SOFA = Sequential Organ Failure Assessment.

### Statistical analysis

Data were analyzed using SPSS version 25.0 (IBM Corp., Armonk, NY, USA). Continuous variables were expressed as mean ± standard deviation (SD), and categorical variables were expressed as number (n) and percentage (%). Normality of data distribution was assessed using the Shapiro-Wilk test. For comparisons between independent groups, the Independent samples t-test or One-Way Analysis of Variance (ANOVA) test were used when parametric test assumptions were met; otherwise, the Mann-Whitney U test or Kruskal-Wallis test were used.

For repeated measures comparisons (dependent groups), Repeated Measures ANOVA was used when parametric assumptions were met; otherwise, the Friedman test was used. Spearman correlation analysis was used to determine correlations between continuous variables. In all analyses, a *p* value < 0.05 was considered statistically significant.

For each analysis, the statistical tests applied were selected according to data structure and study design: comparisons between independent groups were performed using the independent-samples t test or the Mann–Whitney U test (e.g., Table 2), whereas agreement between PEEP selection methods across dependent PEEP steps was assessed using Cohen’s kappa statistics (Tables 3, 4 and 5).

**Table 2:** Hemodynamic and lung mechanics parameters according to PEEP transition steps.

Parameter	Step A High (*n* = 16)	Step A Low (*n* = 14)	*P*	Step B High (*n* = 15)	Step B Low (*n* = 15)	*P*	Step C High (*n* = 16)	Step C Low (*n* = 14)	*P*
Systolic BP (mmHg)	109 (33) [84–117]	99.5 (23) [95–118]	0.285	106 (36) [93.75–129.75]	105 (25) [95.75–120.75]	0.439	109 (34) [100.25–134.25]	108 (30.5) [94.5–125]	0.593
Heart Rate (beats/min)	104.5 (33.5) [83–116.5]	92 (19.7) [85–104.7]	0.109	92 (32) [86–118]	98 (20) [87.5–107.5	0.796	98 (29.5) [87.5–117]	98 (24.5) [86.5–111]	0.593
Mean Arterial Pressure (mmHg)	76.5 (28) [67–95]	70 (14.2) [68.4–82.6]	0.593	77 (25) [69–94]	77 (9) [69.75–78.75]	0.782	79.5 (16.5) [72.75–89.25]	73.5 (21.5) [66.75–99.2]	0.593
Static Compliance (mL/cmH_2_O)	37 (26.5) [30–56.5]	37 (8) [37–45]	0.593	52 (25) [45.75–70.75]	42 (12) [41–53]	0.197	61.5 (21.2) [43–54.2]	52.5 (23.7) [44–67.7]	0.782
Driving Pressure (cmH_2_O)	14 (6.7) [9–15.7]	14.5 (5) [10–15]	0.782	9 (6) [7–13]	11 (5) [8.75–13.75]	0.796	7.5 (4.7) [6–10.7]	10.5 (3.7) [7–10.7]	0.248
PaO_2_/FiO_2_	412.5 (121.7) [340.8–462.5]	315 (169.3) [266–435.3]	0.109	417 (165) [337.25–502.25	350 (187) [238.75–425.75]	0.197	418.5 (125.2) [317–442,2]	307 (124.2) [236.25–360.45]	0.008
EELV (mL)	3149.5 (1969.5) [1414.5–3383]	1874.5 (1892) [1152–3044]	0.033	1435 (1321) [1394–2715]	2146 (1550) [1156–2706]	0.439	2827.5 (2044.7) [1112.5–3157.2]	1387 (2498.7) [759.5–3258.2]	0.033

Values are presented as median (interquartile range) [IQR: 25th–75th percentiles]. Patients were classified as high or low recruiters within each PEEP transition step based on the median R/I ratio for that step. BP = blood pressure; PEEP = positive end-expiratory pressure; EELV = end-expiratory lung volume.

**Table 3:** Patient distribution in step A according to R/I ratio and driving pressure.

n = 30		PEEP Selected According to Driving Pressure		
		15 cmH_2_O PEEP	20 cmH_2_O PEEP	
PEEP Selected According to R/I	15 cmH_2_O PEEP	14 (48.3%)	0 (0%)	Kappa value 0.059 Kappa *p* value 0.341
	20 cmH_2_O PEEP	15 (51.7%)	1 (100%)	

Step A. Distribution of Patients (%) by PEEP Selection Method (R/I Ratio vs. Driving Pressure Criterion) for the 15-to-20 cmH_2_O PEEP Transition Level. Values are presented as n (%). PEEP = Positive End-Expiratory Pressure, R/I = Recruitment/Inflation ratio.

**Table 4:** Patient distribution in Step B according to R/I ratio and driving pressure.

n = 30		PEEP Selected According to Driving Pressure		
		10 cmH_2_O PEEP	15 cmH_2_O PEEP	
PEEP Selected According to R/I	10 cmH_2_O PEEP	15 (50%)	0 (0%)	The kappa value could not be calculated
	15 cmH_2_O PEEP	15 (50%)	0 (0%)	

Step B. Distribution of Patients (%) by PEEP Selection Method (R/I Ratio vs. Driving Pressure Criterion) for the 10-to-15 cmH_2_O PEEP Transition Level. Values are presented as n (%). PEEP = Positive End-Expiratory Pressure, R/I = Recruitment/Inflation ratio.

**Table 5:** Patient distribution in step C according to R/I ratio and driving pressure.

n = 30		PEEP Selected According to Driving Pressure		
		5 cmH_2_O PEEP	10 cmH_2_O PEEP	
PEEP Selected According to R/I	5 cmH_2_O PEEP	12 (52.2%)	2 (28.6%)	Kappa value 0.163 Kappa *p* value 0. 273
	10 cmH_2_O PEEP	11 (47.8%)	5 (71.4%)	

Step C. Distribution of Patients (%) by PEEP Selection Method (R/I Ratio vs. Driving Pressure Criterion) for the 5-to-10 cmH_2_O PEEP Transition Level. Values are presented as n (%). PEEP = Positive End-Expiratory Pressure, R/I = Recruitment/Inflation ratio.

In our study, we aimed to investigate the relationship between driving pressure and the Recruitment/Inflation ratio at two consecutive PEEP levels for appropriate PEEP selection in patients receiving mechanical ventilation support in the ICU.

## Results

### Baseline characteristics

A total of 30 mechanically ventilated non-ARDS ICU patients were enrolled (Table 1). Their demographic and clinical characteristics closely resemble previously published non-ARDS ICU cohorts undergoing decremental PEEP trials and EELV monitoring^1–4^. The median APACHE II score in our population (22) was comparable to illness severity reported in earlier non-ARDS ventilation studies^13,14^. Before PEEP titration, baseline respiratory system compliance and driving pressure values suggested relatively preserved lung mechanics compared with classic ARDS cohorts^7,15^.

### Hemodynamic and lung mechanics across PEEP steps

Hemodynamic variables (heart rate, systolic arterial pressure, and mean arterial pressure) remained stable across all decremental PEEP transitions, and no step demonstrated clinically relevant cardiovascular change (Table 2). This behavior aligns with prior decremental PEEP and EELV-guided trials, in which short PEEP steps did not induce major acute hemodynamic instability^1,4,5,16^.

Respiratory mechanical parameters changed consistently with decreasing PEEP: driving pressure decreased and static compliance increased from Step A (20→15 cmH_2_O) to Step C (10→5 cmH_2_O) (Table 6). This pattern is compatible with overdistension unloading and movement toward a more favorable portion of the pressure–volume curve, as described in physiological and clinical studies of PEEP-induced lung volume change^4,7,17–20^.

**Table 6:** Driving pressure and static compliance (ml/cmH_2_O) at different PEEP levels (cmH_2_O).

n = 30	Arithmetic Mean ± SD	Median (IQR)	Min–Max
Driving pressure			
20cmH_2_O PEEP	13,31 ± 3,34	12 (6)	6–18
15 cmH_2_O PEEP	10,65 ± 3,11	10 (5)	6–18
10 cmH_2_O PEEP	9,08 ± 2,77	9 (4)	5–15
5 cmH_2_O PEEP	8,92 ± 2,91	8 (5)	5–15
0 cmH_2_O PEEP	11,42 ± 4,33	10,50 (9)	6–20
Intragroup *p* value	< 0,001		
Static compliance (ml/cmH_2_O)			
20 cmH_2_O PEEP	41,65 ± 13,01	39,5 (13)	20–77
15 cmH_2_O PEEP	52,81 ± 15,60	49,5 (22)	30–94
10 cmH_2_O PEEP	60,73 ± 16,33	63,5 (23,75)	35–100
5 cmH_2_O PEEP	67,80 ± 29,19	64 (45,25)	33–150
0 cmH_2_O PEEP	56,46 ± 20,61	56 (39)	25–95
Intragroup *p* value	< 0,001		

Driving pressure and compliance values at different peep levels. Min: minimum, Max: maximum, PEEP = Positive End-Expiratory Pressure.

### R/I ratio classification across PEEP steps

The R/I ratio classified patients as high or low recruiters at each PEEP step (Table 2). A heterogeneous recruitability pattern emerged, consistent with previous R/I studies in ARDS and COVID-19 ARDS populations, which similarly demonstrated large inter-patient variability in recruitment response to PEEP^8^. During the lowest PEEP transition (10→5 cmH_2_O), high recruiters showed greater improvement in EELV and PaO₂/FiO₂ than low recruiters, reflecting stronger aeration response at lower lung volumes^8,12,21,22^.

### Agreement between R/I ratio and driving pressure-guided PEEP selection

Agreement between R/I ratio-based classification and PEEP level selection by driving pressure minimization was evaluated using Cohen’s kappa (Tables 3, 4 and 5). In Step A, occasional concordance occurred between methods but did not reach statistical significance. In Step B, comparison was not feasible because 15 cmH₂O was never selected as optimal by driving pressure. In Step C, partial overlap appeared, but overall agreement remained weak and statistically non-significant^7,8,17–24^.

### Relationship of the R/I ratio with lung mechanics and oxygenation

Across steps, patients defined as high recruiters by the R/I ratio demonstrated higher static compliance, larger EELV and higher PaO_2_/FiO_2_ than low recruiters, especially during the lowest PEEP transition. This suggests that R/I may reflect distension capacity rather than pure recruitment, supporting prior work questioning whether ΔEELV represents reopening of collapsed alveoli or inflation of aerated lung^17–25^.

## Discussion

Lung-protective ventilation principles—low tidal volume, plateau pressure limitation, and careful PEEP titration—are fundamental in mechanical ventilation for both ARDS and non-ARDS patients^13,14^. Within this framework, driving pressure has emerged as a critical mechanical determinant associated with survival, and minimizing driving pressure has been proposed as a pragmatic target during PEEP titration^7^. Meanwhile, the recruitment-to-inflation (R/I) ratio was developed as a bedside index of recruitability, providing volume-based information on pressure responsiveness^8^.

The primary finding of this study is that PEEP levels suggested by the R/I ratio did not show statistically significant agreement with PEEP levels determined by driving pressure minimization in non-ARDS mechanically ventilated ICU patients. Although individual instances of matching PEEP recommendations occurred—mainly during the lowest PEEP transition—the overall concordance remained weak and non-significant^7,8,17–24^.

Our data suggest that R/I and driving pressure capture different physiological dimensions. Driving pressure reflects the relationship between tidal volume and functional lung size and is strongly linked to ventilator-induced lung injury and mortality^7,13,14,18–26^. Conversely, the R/I ratio quantifies volume change with pressure shifts and was initially validated against multiple recruitment assessments^8,17^. In our cohort, high R/I values correlated with greater EELV, higher compliance and improved oxygenation, consistent with prior ARDS-based data^8,12,21,22^. However, these improvements did not consistently align with the PEEP level that minimized driving pressure, indicating that R/I should not guide PEEP selection alone.

Previous work indicates that ΔEELV may overestimate true recruitment, particularly in patients with preserved mechanics, because inflation of open units can be misinterpreted as reopening of collapsed tissue^17,23^. Gattinoni and colleagues described true recruitment as re-aeration of previously collapsed lung^24^. In this context, our findings support the interpretation that R/I in non-ARDS patients may reflect inflation dynamics rather than classic recruitment^17–25^.

Hemodynamic stability across steps strengthens our analysis. Unlike some studies in ARDS and COVID-19, where lower PEEP was associated with improved blood pressure, we observed no significant arterial pressure change, possibly due to non-ARDS status and short step intervals^4–6,16–22^.

Clinically, although R/I provided meaningful physiology, it did not replace driving pressure for identifying the most lung-protective PEEP. These results support multimodal PEEP titration approaches integrating mechanics, EELV response, and oxygenation^25,26^.

### Limitations

This study has several limitations that should be acknowledged. First, the R/I ratio cut-off was defined retrospectively using the median value calculated within the study population. While this approach is consistent with prior studies, it limits immediate bedside applicability and may reduce external validity. Second, end-expiratory lung volume measurements were performed once at each PEEP level for safety reasons, and measurement variability could have influenced the calculated R/I ratios. Third, airway opening pressure was not measured, which precludes assessment of its potential impact on the interpretation of recruitability during PEEP transitions. Finally, the study population consisted exclusively of non-ARDS mechanically ventilated patients; therefore, the findings may not be generalizable to patients with established ARDS or severe lung heterogeneity.

## Conclusion

In conclusion, although occasional concordance was observed, PEEP selection guided by the R/I ratio did not significantly agree with driving pressure–based PEEP selection in non-ARDS mechanically ventilated patients. While the R/I ratio may provide complementary physiological insight, our findings do not support its use as a standalone tool for individualized PEEP titration. Further prospective studies incorporating predefined cut-offs and clinically relevant outcomes are needed to clarify the role of the R/I ratio in bedside ventilatory management.

What is already known

1. Positive end-expiratory pressure (PEEP) titration is crucial for preserving lung mechanics and physiology in mechanically ventilated patients. End-Expiratory Lung Volume (EELV) and compliance can guide this process. Based on these parameters, the Recruitment/Inflation ratio (R/I ratio) has been utilized to estimate lung recruitability.
2. Methods such as using driving pressure have been described in the literature for determining appropriate PEEP; however, the optimal method remains undetermined.

What this study adds

1. This study investigated the relationship between the R/I ratio and PEEP selection based on driving pressure. Based on our results, sufficient and robust evidence to support the standalone clinical use of the R/I ratio for this purpose was not found.
2. The agreement between PEEP selection guided by driving pressure and PEEP selection guided by the R/I ratio was not statistically significant in our cohort.
3. Although previous studies have found the R/I ratio to be correlated with various parameters, it is insufficient for determining appropriate PEEP on its own.

## Supplementary Information

Below is the link to the electronic supplementary material.

Supplementary Material 1

## Data Availability

The datasets used and analyzed during the current study are available from the corresponding author on reasonable request.

## Abbreviations

COVID-19: Coronavirus-19 disease
M: Male
EELV: End-expiratory lung volume (End-expiratory lung volume)
FiO_2_: Fraction of inspired oxygen (Percentage of oxygen in the air inhaled)
FRK: Functional residual capacity
IQR: Interquartile range (Interquartile range)
Min–Max: Minimum–maximum
NMBW: Nitrogen multiple breath washout
PaCO_2_: Partial arterial carbon dioxide pressure
PaO_2_: Partial arterial oxygen pressure
PEEP: Positive end-expiratory pressure
PIP: Peak airway pressure
RI: Recruitment to inflation ratio
SpO_2_: Peripheral oxygen saturation
SD: Standard deviation
BMI: Body mass index
VT: Tidal volume
ICU: Intensive care unit
