# Supplementary material for: Association between driving pressure and recruitment-to-inflation ratio in personalized PEEP management at the bedside
Source: Sci Rep. 2026 Jan 19;16:5711. doi: 10.1038/s41598-026-36300-z (PMC12891550; doi:10.1038/s41598-026-36300-z)
Supplement: Supplementary file 1 — Supplementary Material 1 [file 41598_2026_36300_MOESM1_ESM.docx]

**Supplementary Figure S1**

**Time and Location:**

July 2023 – July 2024

**Participants**

30 patients (aged 18–86 years)

Inclusion and exclusion criteria applied

**Recruitment Maneuver**

20 cmH₂O PEEP for 30 seconds

Decremental PEEP: 20–15–10–5 cmH₂O

**R/I Ratio Calculation**

V_Est. = ΔEELV – (ΔPEEP × Compliance at low PEEP)

C_Est = V_Est. / ΔPEEP

R/I = C_Est / Compliance at low PEEP

**PEEP Selection**

Optimal PEEP lowest driving pressure

Ventilated for 24 hours

**
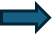
**

**Stepping**

Step A: 20–15 cmH₂O Step B: 15–10 cmH₂O Step C: 10–5 cmH₂O

High Recruiters: R/I ≥ cut-off → High PEEP

Low Recruiters: R/I < cut-off → Low PEEP

Ventilated for 24 hours

**Analysis**

Correlation between PEEP by driving pressure and R/I ratio

Fıgure 1. Flowchart. PEEP= Positive End-Expiratory Pressure, V_Est=Estimated recruited volume, EELV=End-expiratory lung volume, C_Est =Normalize recruited volume estimated, R/I= Recruitment/inflation ratio
